# Cytotoxicity and genotoxicity of stilbene derivatives in CHO-K1 and HepG2 cell lines

**DOI:** 10.1590/1678-4685-GMB-2016-0214

**Published:** 2017-07-10

**Authors:** Cassia Suemi Mizuno, Winnifred Ampomaah, Fernanda Ribeiro Mendonça, Gabriela Carvalho Andrade, Ariel Maria Nazaré da Silva, Mirian Oliveira Goulart, Raquel Alves dos Santos

**Affiliations:** 1 Department of Pharmaceutical Sciences, University of New England - College of Pharmacy, Portland, ME, USA; 2 Universidade de Franca, Franca, SP, Brazil

**Keywords:** stilbene, cytotoxicity, genotoxicity, micronucleus assay

## Abstract

The cytotoxicity and genotoxicity of the stilbenes (*E*)-methyl-4-(3-5-dimethoxystyryl)benzoate (ester), (*E*)-4-(3-5-dimethoxystyryl)aniline (amino), (*Z*)-1,3-dimethoxy-5-(4-methoxystyryl)benzene (*cis*-TMS) and (*E*)-1,3-dimethoxy-5-(4-methoxystyryl)benzene (*trans*-TMS) were investigated in this work. Structural modifications of resveratrol, a naturally occurring stilbene, have been previously performed, including the replacement of hydroxyl by different functional groups. Such modifications resulted in significant improvement of target-specific effects on cell death and antiproliferative responses. The parameters were evaluated using XTT assay, clonogenic survival assay and the cytokinesis-block micronucleus assay in CHO-K1 and HepG2 cell lines. The results showed that *cis*-TMS is approximately 250-fold more cytotoxic than the amino and ester, and 128-fold more cytotoxic than *trans*-TMS. When genotoxicity was evaluated, only the *trans*-TMS did not significantly increase the frequency of micronucleus (MN). While the *cis*-TMS induced a mean of 5.2 and 5.9 MN/100 cells at 0.5 μM in CHO-K1 and HepG2, respectively, the amino and ester induced 3.1 and 3.6 MN/100 cells at 10 μM in CHO-K1, respectively, and 3.5 and 3.8 in HepG2. *Trans-*TMS is genotoxic only in HepG2 cells. Based on these results, the *cis*-TMS was the most cytotoxic and genotoxic compound in both cell lines.

## Introduction

Stilbenes comprise a class of plant-derived secondary metabolites that are produced in response to fungal infections (Flamini *et al.*, 2016). Resveratrol is one of the most investigated stilbenes and has been found in many species of plants that are part of our diet such as grapes, peanuts, pomegranade and berries. Resveratrol became the focus of intense research after its presence in wine was associated with the French paradox in which a wine-drinking population with a high intake of saturated fat showed low incidence of cardiovascular diseases. The cardioprotective effects of this compound occur through different mechanisms including inhibition of platelet aggregation (Shen *et al.*, 2007), vasorelaxation effects by upregulating NOS expression (Wallerath *et al.*, 2002), and inhibition of LDL oxidation and subsequent atherosclerosis (Belguendouz *et al.*, 1997). Resveratrol is also a scavenger of H_2_O_2_, a reactive oxygen species involved in oxidative stress (Ungvari *et al.*, 2007).

**Figure 1:**
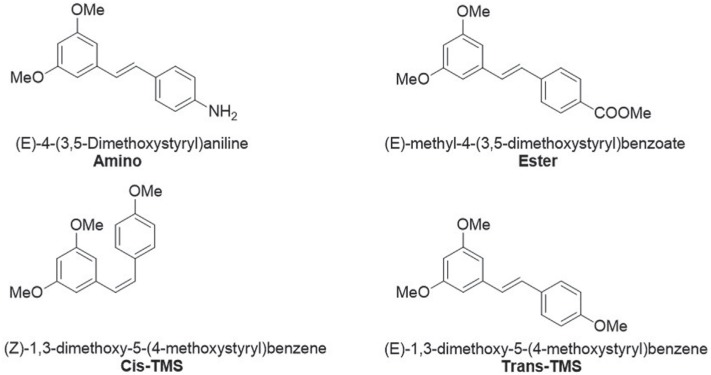
Chemical structure of stilbenes.

Other naturally occurring stilbenes with similar therapeutic potential include pterostilbene and piceatannol with anticancer (Seyed *et al.*, 2016), anti-virus (Lee *et al.*, 2016) antioxidant (Mikstacka *et al.*, 2010), anti-inflammatory (Mikstacka *et al.*, 2010), and lipid-lowering (Rimando *et al.*, 2005) effects among others. Stilbenes reportedly inhibited the growth of several cancer cell lines such as colon, breast, prostate, pancreas, melanoma, lung and others. This effect is elicited through different mechanisms including cell cycle arrest and apoptosis induction via caspase activation (Aggarwal *et al.*, 2004). Several animal studies support the evidence provided by the *in vitro* assays. Resveratrol prevented the formation of colon and intestinal tumors by 70% in Min mice (Schneider *et al.*, 2001). 3,5,4'trimethoxystilbene (*trans*-TMS, Figure 1), a natural compound, significantly reduced tumor growth in Colo 205 xenograft model of colon cancer (Pan *et al.*, 2008) and pterostilbene reduced the formation of azoxymethane-induced colonic aberrant crypt foci (ACF) (Suh *et al.*, 2007). In a work by Paul *et al.* (2010), two stilbene derivatives (amino and *trans*-TMS, Figure 1) significantly decreased tumor volume and weight in HT-29 xenografts model of colon cancer. Based on the promising and pleiotropic effects of the stilbenes, several clinical trials have investigated the effects of resveratrol and pterostilbene in patients with cardiovascular diseases, type 2 diabetes, chronic obstructive pulmonary disease and metabolic syndrome.

With the consideration of stilbenes as potential drug candidates, it is very important to determine the toxicity of these compounds. The genotoxicological analysis is of fundamental importance during toxicological evaluation of therapeutic drug candidates. Genotoxic compounds are capable of interacting with the DNA molecule, leading to genetic damage in essential regions for cycle-control and apoptosis, with the subsequent initiation of neoplastic processes (Santos and Takahashi 2008). Genotoxicity tests for screening substances (*e.g.* drug candidates, medicinal plant extract, chemical substance, etc) officially approved by the Organization for Economic Co-operation and Development (OECD) guidelines include *in vitro* micronucleus test to detect both clastogenic and aneugenic effects of potential drug candidates (Fenech, 2000).

The cytotoxicity and genotoxicity of the stilbenes (*E*)-methyl-4-(3-5-dimethoxystyryl)benzoate (ester), (*E*)-4-(3-5-dimethoxystyryl)aniline (amino), (*Z*)-1,3-dimethoxy-5-(4-methoxystyryl)benzene (*cis*-TMS) and (*E*)-1,3-dimethoxy-5-(4-methoxystyryl)benzene (*trans*-TMS) (Figure 1) have not been reported in non-tumoral cell lines. Therefore, in the present study we investigated the cytotoxicity and genotoxicity of these stilbene derivatives using the CHO-K1 and HepG2 cell lines.

## Material and Methods

### Chemicals and reagents

Dimethyl sulfoxide (DMSO, CAS 67-68-5), cytochalasin B (CytB, CAS 14930-96-2), doxorubicin hydrochloride (DOX, CAS 25316-40-9) and reagents for cell culture and micronucleus tests were purchased from Sigma-Aldrich (St. Louis, USA). Cell Proliferation Kit (XTT) was purchased from Roche (Mannheim, Germany). All solvents were anhydrous and all reactions were performed under an inert atmosphere, unless aqueous. All round-bottom flasks were kept dried in an oven under 100 °C. Flash chromatography used to purify the compounds was done using a Teledyne Isco CombiFlash and LC-MS were obtained from Waters Micromass Quattro LC. The purity of all stilbenes tested was above 95%.

### General procedure for the synthesis of stilbene derivatives

To a cold solution (–78 °C) of phosphonium salt (1.0 equivalent) in THF was added *n*-butyllithium (1.6 mol in hexanes, 1.0 equivalent) and the resulting solution was stirred under inert atmosphere for 2 h. A solution of aldehyde (1.0 equivalent) in THF was added dropwise, and the mixture was stirred for 12 h at room temperature. The resulting suspension was poured into water and extracted with dichloromethane. The organic phase was combined and dried over MgSO_4_, and concentrated under reduced pressure. The crude product was purified through automated flash purification elution with hexanes/ethyl acetate.

(E)-Methyl 4-(3,5-dimethoxystyryl)benzoate (ester): Reaction of (3,5-dimethoxybenzyl) triphenylphosphonium 4 (300 mg, 0.608 mmol) and methyl 4-formylbenzoate (100 mg, 0.608 mmol) afforded the ester as a white solid: 32 mg (17%). ^1^H NMR (CDCl_3_, 300 MHz): δ 3.83 (s, 6H); 3.92 (s, 3H); 6.42 (s, 1H); 6.68 (s, 2H); 7.04–7.16 (m, 2H); 7.53 (d, 2H, *J* = 8.4 Hz); 8.01 (d, 2H, *J* = 8.4 Hz). ^13^C NMR (CDCl_3_, 75 MHz): δ 52.1, 55.4 (2C), 100.6, 104.9 (2C), 126.4 (2C), 128.1, 129.0, 130.1 (2C), 131.3, 138.8, 141.7, 161.1 (2C), 166.9. LCMS m/z 299.28. (M+H)^+^

(E)-4-(3,5-Dimethoxystyryl) aniline (amino). Reaction of (3,5 dimethoxybenzyl) triphenylphosphonium (300 mg, 0.608 mmol) and 4-nitrobenzaldehyde (92 mg, 0.608 mmol) afforded 1,3-dimethoxy-5-(4-nitrostyryl)benzene. A solution of the latter (100 mg, 0.35 mmol) in acetone/water (10:5 mL) was heated to 50 °C for 30 min. Sodium dithionite (1526 mg, 8.76 mmol) was slowly added and the mixture was heated to reflux for 1 h. After cooled to room temperature the mixture was poured into water and extracted with ethyl acetate. The organic phase was combined and dried over MgSO_4_, and solvent was removed under reduced pressure. The crude mixture was purified using automated flash chromatography eluting with hexanes giving the amine as a yellow powder: 29 mg (25% yield) ^1^H NMR (CDCl_3_, 300 MHz): δ 3.82 (s, 6H); 6.36 (s, 1 H); 6.63–6.69 (m, 4H); 6.85 (d, 1H, *J* = 16.2 Hz); 7.00 (d, 1H, *J* = 16.2 Hz); 7.33 (d, 2H, *J* = 8.4 Hz). δ ^13^C NMR (CDCl_3_, 75 MHz): δ 55.4 (2C), 99.4, 104.3 (2C), 115.2 (2C), 125.1, 127.8, 127.9, 129.3, 140.1, 146.4, 161.0 (2C). LCMS m/z 256.23 (M+H)^+^

1,3-dimethoxy-5-(4-methoxystyryl)benzene- cis and trans-TMS. Reaction of (3,5-dimethoxybenzyl)triphenylphosphonium (300 mg, 0.608 mmol) and 4-methoxybenzaldehyde (75 μL, 0.608 mmol) afforded the cis and trans-TMS. Trans-TMS as a white solid: 15 mg (9%). ^1^H NMR (CDCl_3_, 300 MHz): δ 3.83 (s, 9H); 6.37 (t, 1H, *J* = 2.1 Hz); 6.65 (d, 2H, *J* = 1.8 Hz); 6.87-6.89 (m, 2H); 6.92 (d, 1H, *J* = 5.1 Hz); 7.04 (d, 1H, *J* = 16.2 Hz); 7.45 (d, 2H, *J* = 6.9 Hz). ^13^C NMR (CDCl_3_, 75 MHz): δ 55.2 (3C), 99.7, 104.2 (2C), 114.2 (2C), 126.5, 127.8 (2C), 129, 130, 139.6, 159.4, 161.0 (2C). LC-MS *m/z* 270.19. (M+H).^+^ Cis-TMS as a viscous liquid: 62 mg (37%). ^1^H NMR (CDCl_3_, 75 MHz): δ 3.68 (s, 6H); 3.78 (s, 3H); 6.34 (t, 1H, *J* = 2.4 Hz); 6.44–6.46 (m, 3H); 6.55 (d, 1H, *J* = 12.3 Hz); 6.78 (d, 2H, *J* = 8.7 Hz); 7.24 (d, 2H, *J* = 8.4 Hz). ^13^C NMR (CDCl_3_, 75 MHz): δ 55.3 (3C), 99.7, 106.7 (2C), 113.6 (2C), 128.7, 129.6, 130.2, 130.3 (2C), 139.5, 158.8, 160.6 (2C). LC-MS *m/z* 271.28 (M+H)^+^.

### Cell culture and treatment conditions

CHO-K1 and HepG2 cells were obtained from the Cell Bank of Rio de Janeiro (BCRJ code 0069 and 0103, respectively) and cultured in complete medium containing DMEM+F10 nutrient mixture (1:1, v/v), supplemented with fetal bovine serum (10%, v/v) and penicillin/streptomycin stabilized solution (10 mL/L). Cell cultures were incubated at 37 °C with atmosphere saturated with 5% of CO_2_. All experiments were conducted between the 3^rd^ and the 8^th^ passages and three experimental repetitions were performed.

The ester, amino, *cis*-TMS and *trans–*TMS were dissolved in DMSO in a stock concentration of 0.1 M. The final concentration of DMSO during treatments did not exceed 0.1% (v/v) in cell cultures. Doxorubicin (DOX) was dissolved in ultrapure sterile water in a stock solution of 900 μM. All reagents were dissolved prior to use and protected from light. For all experiments, cells were submitted to 24 h treatment with different concentrations of the tested substance and DOX was used as positive control in CHO-K1 cells. Benzo(a)pyrene [B(a)P] at 25 μM was used as positive control in HepG2 cells.

### Cell viability assay – XTT

To determine cell viability, 10^4^ cells/well were seeded on a 96-well plate. After 24 h the cells were treated with ester, amino, *cis*-TMS and *trans–*TMS in a concentration ranging from 7.8 μM to 1000 μM and incubated for an additional 24 h. The plates were washed twice in phosphate buffer saline (PBS 1X) and incubated for 4 h at 37 °C with DMEM without phenol red and supplemented with the reagents of the Cell Proliferation Kit (XTT) as recommended by the manufacturer. Total absorbance was measured at 492 and 690 nm (reference) using a microplate reader (ASYS, Eugendorf, Salzburg, Austria). Results of total absorbance were considered directly proportional to the number of viable cells as a percentage of the negative control (100% of cell viability).

### Clonogenic survival assay

The clonogenic assay was performed according to Franken *et al.* (2006). Briefly, 200 cells/well were plated in 6 well plates containing 3 mL of complete medium. After 4 h of incubation at 37 °C, the cells were treated with different concentrations of the testing stilbenes for 24 h, then each well was washed with PBS 1X and complete medium was added. The cells were allowed to grow for 7–14 days at 37 °C and 5% of CO_2_, when colonies were visible. The colonies formed were fixed with methanol/acetic acid/water (1:1:8 v/v/v) and stained with Giemsa 5% (v/v) in Sorensen phosphate buffer (pH 6.8). The colonies were counted, and the cell survival fraction was calculated as percent colonies relative to the untreated control.

### Micronucleus test

For the micronucleus test, 10^6^ cells were seeded in 25 mm^2^ culture flasks containing complete medium. Cell cultures were allowed to grow for 24 h, then treated with different concentrations of the stilbene derivatives. Simultaneously, cytochalasin B (6 μg/mL) was added to each cell culture to inhibit cytokinesis. After 24 h of incubation, cells were harvested with a hypotonic treatment (KCl 0.075 M) and fixed twice with methanol:acetic acid (3:1). Thereafter, the cell suspension was smeared on pre-cleaned microscope slides and air-dried. Staining was performed during 15 min with Giemsa at 5% in Sorensen Buffer (v/v). Microscope slides were washed in tap water and allowed to dry at room temperature overnight. The frequency of micronucleus (MN), frequency of binucleated cells with micronucleus (BCMN) and frequency of nucleoplasmic bridges (NB) were obtained by the analysis of 1000 binucleated cell/culture/treatment as previously described by Fenech (2000). A total of 500 cells/culture/treatment were analyzed to determine the Nuclear Division Index (NDI) by scoring cells with 1, 2, 3 or 4 nuclei and using the formula: NDI= M1+2(M2)+3(M3)+4(M4)/N, where, M1–M4 stand for the number of cells with 1–4 nuclei and N is the total number of viable cells analyzed.

### Statistical analysis

Statistical analysis of experimental data was performed using the software GraphPad Prism 5.0. The IC_50_ was calculated by linear regression analysis and the comparative analysis among experimental groups was done with ANOVA, followed by Tukey's post-hoc test when significant differences among treatments were found. The significance was set at p < 0.05 and the results were reported as means and standard deviations (SD) of three independent experimental samples assayed in triplicates.

## Results

### The cytotoxicity of stilbene derivatives in CHO-K1 and HepG2 cells

**Figure 2:**
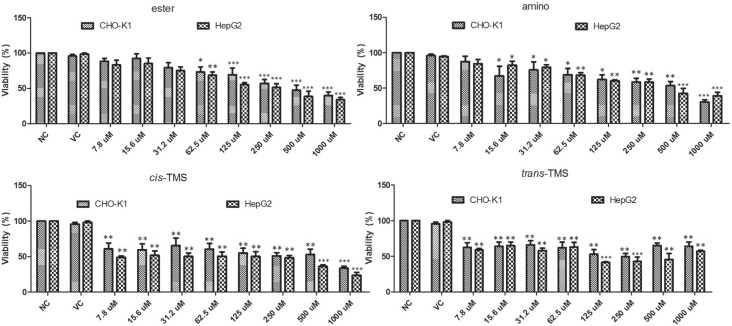
Mean values of cell viability obtained by XTT assay in CHO-K1 and HepG2 cell lines after 24 h of treatment with different concentrations of ester, amino, *cis*-TMS and *trans*-TMS stilbenes. NC: negative control; VC: vehicle control (DMSO 0.1%). *p < 0.05,**p < 0.01 and ***p < 0.001 compared to negative control.

The cytotoxicity of the stilbenes was determined by the XTT assay. Cells were treated with different concentrations of the tested stilbenes for 24 h and the results are presented in Figure 2. Cell viability was significantly reduced (p < 0.05) by the ester at 62.5 μM (73.3 and 68.6% in CHO-K1 and HepG2, respectively). Significant reduction in cell viability by the amino was observed at 15.6 μM (67.2%) and 62.5 μM (68.2%) in CHO-K1 and HepG2, respectively, and at all concentrations of the *cis*-TMS and *trans–*TMS in both cell lines. At the lowest concentration (7.8 μM) of the *cis*- and *trans*-TMS, the cell viability was 60.7% and 62.5%, respectively in CHO-K1, and 49% and 59.1% in HepG2 (p < 0.05). Tukey's test demonstrated that the ester and amino induced a concentration-dependent reduction in cell viability, differently from what was observed for the *cis* and *trans*-TMS in both cell lines.

**Figure 3:**
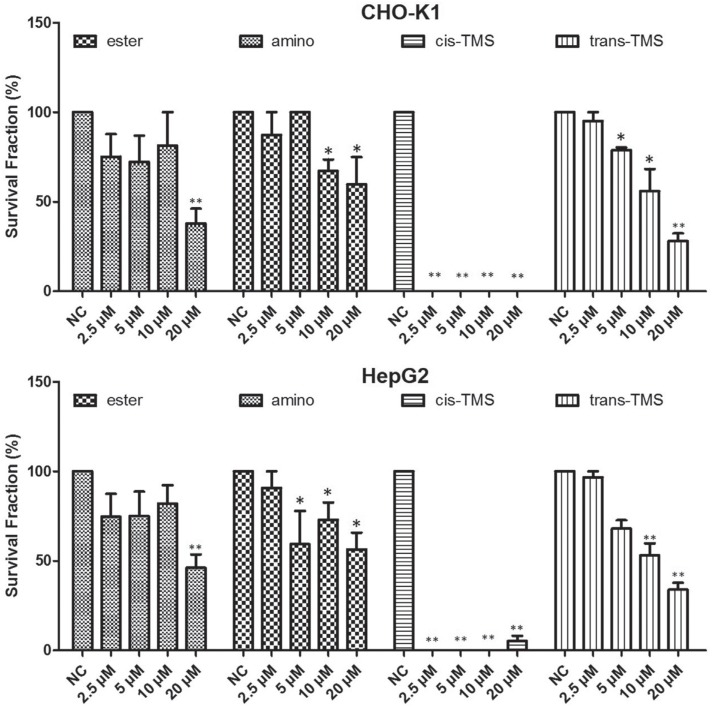
Clonogenic assay showing the survival fraction of CHO-K1 and HepG2 cell lines after 24 h treatment with different concentrations of ester, amino, *cis*-TMS and *trans*-TMS stilbenes. Cells were treated for 24 h and allowed to grow during seven consecutive days. NC: negative control. *p < 0.05 and **p < 0.01 compared to negative control.

**Figure 4:**
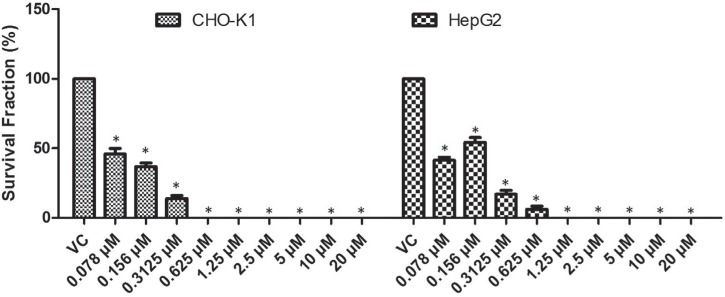
Clonogenic assay showing the survival fraction of CHO-K1 and HepG2 cell lines after 24 h treatment with different concentrations of *cis*-TMS. Cells were treated for 24 h and allowed to grow during seven consecutive days. NC: negative control. *p < 0.05 compared to vehicle control.

Subsequently, the long-term cytotoxic effects of stilbene derivatives were evaluated by the clonogenic survival assay. Based on the XTT results, all stilbenes were tested in concentrations ranging from 2.5 μM to 80 μM. However, no stilbene could form countable colonies at 40 and 80 μM (data not shown). According to the results exhibited in Figure 3, in CHO-K1, the ester significantly reduced (p < 0.05) the frequency of survival fraction at 20 μM (37.8%) and the amino at 10 and 20 μM (67.1 and 59.6% respectively). Similarly, in HepG2, 20 μM ester significantly reduced the survival fraction (46.1%) while significant response to amino treatment was observed at 5 μM (59.3%). The *trans*-TMS showed a significant concentration-dependent reduction in the frequency of survival fraction in both cell lines. Interestingly, the *cis-*TMS showed much higher cytotoxicity than the other tested stilbenes and killed 100% of the cells in all concentrations tested in CHO-K1 cells and at 2.5, 5 and 10 μM in HepG2. Therefore, this compound was tested at lower concentrations than the other stilbenes (Figure 4). The survival fraction was significantly reduced by the *cis*-TMS to 45.8, 36.8 and 13.9% at 0.078, 0.156 and 0.3125 μM, respectively in CHO-K1 cells and 41.3, 54.3, 16.9 and 6.2% at 0.078, 0.156, 0.3125 and 0.625 μM in HepG2.

### Genotoxicity of stilbene derivatives detected by cytokinesis-blocking micronucleus assay (CBMN)

**Figure 5:**
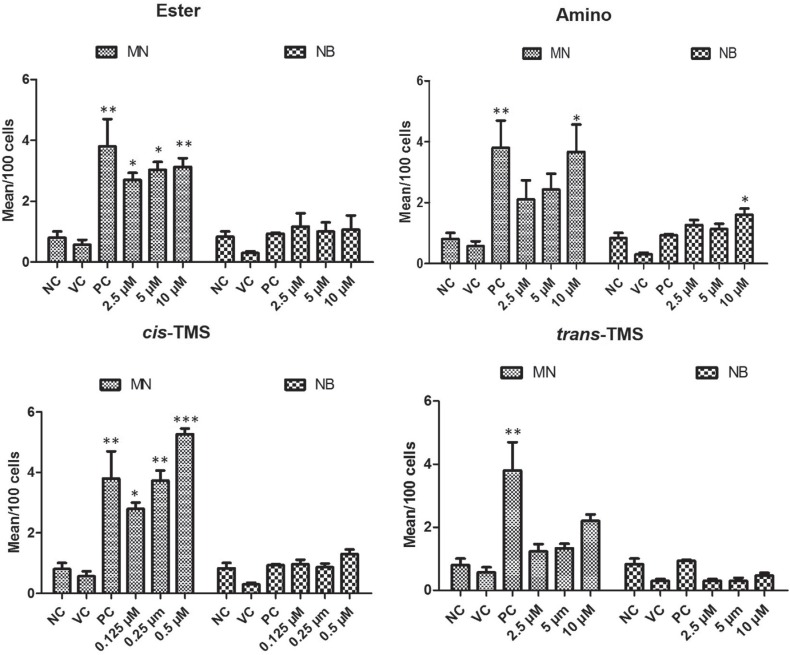
MN assay showing the analysis of genotoxicity in CHO-K1 cells treated with different concentrations of ester, amino, *cis*-TMS and *trans*-TMS stilbenes. NC: negative control; VC: vehicle control (DMSO 0.1%); PC: positive control (DOX 0.5 μM in CHO-K1 and B(a)P 25 μM in HepG2). *p < 0.05,**p < 0.01 and ***p < 0.001 compared to vehicle control.

For the genotoxicity analysis, CBMN assay was performed to detect micronucleus frequency (MN) and nuclear bridges (NB) in both CHO-K1 and HepG2 cells. According to the results presented in Figure 5, the ester, amino and *cis*-TMS increased MN frequency in CHO-K1 cells. However, no significant MN frequency (p < 0.05, compared to NC) occurred in cells treated with *trans*-TMS at all concentrations. The ester increased the mean of MN to 2.7, 3 and 3.1 at 2.5, 5 and 10 μM, respectively, in comparison to the NC (Figure 5). Similar effect was observed with the amino only at 10 μM, where 3.6 MN were detected per 100 cell (p < 0.05, Figure 5). All tested concentrations of c*is*-TMS increased the mean of MN in CHO-K1 cells. Even the lowest concentration tested (0.125 μM) increased the mean to 2.8 MN/100 cells (p < 0.05). At the highest concentration (0.5 μM), the frequency of MN was 5.2/100 cells (p < 0.001). Nuclear bridges were only observed in cells treated with the amino at 10 μM.

**Figure 6:**
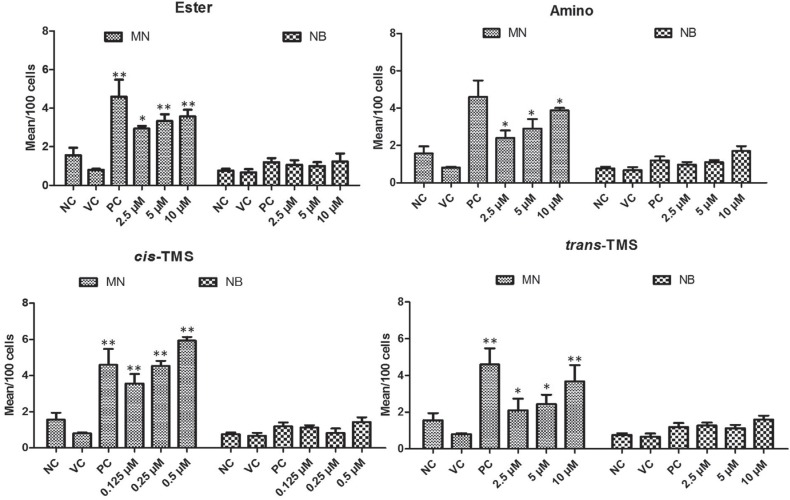
MN assay showing the analysis of genotoxicity in HepG2 cells treated with different concentrations of ester, amino, *cis*-TMS and *trans*-TMS stilbenes. NC: negative control; VC: vehicle control (DMSO 0.1%); PC: positive control (DOX 0.5 μM). *p < 0.05,**p < 0.01 and ***p < 0.001 compared to vehicle control.

The analysis of genotoxicity in HepG2 revealed that all tested stilbenes increased the frequency of MN when compared to the negative control (p < 0.05) (Figure 6). The lowest concentration of *cis*-TMS (0.125 μM) induced a mean of 3.5 MN/100 cells. At 0.5 μM of *cis-*TMS the frequency of MN observed was 6.1/100 cells. Treatments with ester increased the frequency of MN to 2.9 (2.5 μM), 3.3 (5 μM) and 3.5 (10 μM)/100 cells, while amino induced 2.4, 2.9 and 3.8 MN/100 cells at 2.5, 5 and 10 μM, respectively. Interestingly, the frequency of MN was significantly increased by *trans*-TMS treatments in all tested concentrations. No significant induction of nuclear bridges was observed in response to treatments with ester, amino, *cis* and *trans*-TMS in HepG2 cells.

**Figure 7:**
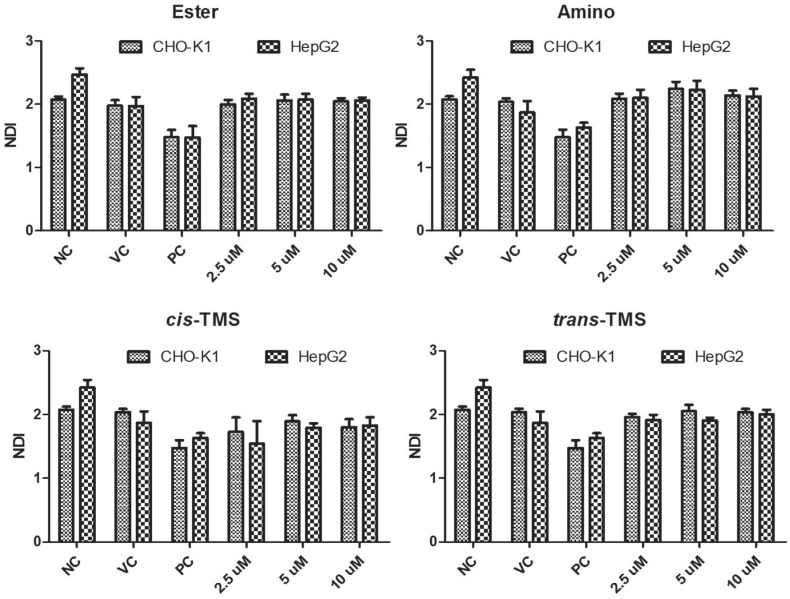
Nuclear Division Index (NDI) obtained in CHO-K1 and HepG2 cell lines treated with different concentrations of ester, amino, *cis*-TMS and *trans*-TMS stilbenes. NC: negative control; VC: vehicle control (DMSO 0.1%); PC: positive control (B(a)P 25 μM).

No alteration in the nuclear division (NDI) was observed in response to any treatment when compared to the NC in both cell lines (p > 0.05, Figure 7).

## Discussion

In the present study, the cytotoxicity and genotoxicity of the ester, amino, *cis*-TMS and *trans*-TMS stilbenes were investigated in CHO-K1 and HepG2 cell lines. CHO-K1 is recommended by the OECD for genotoxicity screening (OECD, 2014). However, HepG2 is more sensitive towards some genotoxins and enables detection of genotoxic carcinogens, which give negative results in other currently used bioassays, suggesting that in some cases, they might be more suitable than cell lines currently used for routine screening (Knasmüller *et al.*, 2004). The cytotoxic (XTT) assay was performed to stablish the non-cytotoxic concentrations of the tested compounds to be used in the evaluation their genotoxic potential. The determination of the non-cytotoxic concentration is important because according to the OECD (2014) MN assay, cell proliferation must occur in both control and treated cultures to assess the extent of chemical-induced cytotoxicity or cytostasis in all of the cell cultures in which micronuclei are detected. Cytotoxicity of stilbene derivatives was determined using both the XTT and clonogenic survival assay, which evaluate the short and long term effects of a drug on cell viability and proliferation, respectively, and confirmed by the analysis of nuclear division index through the MN assay.

According to the XTT assay (Figure 2), the most cytotoxic compounds were the *cis* and *trans*-TMS, showing a significant reduction of cell viability at 7.8 μM in both cell lines. The ester was the least cytotoxic compound with a significant reduction of cell viability observed at 62.5 μM in CHO-K1 and HepG2. The reduction of cell viability may be associated with necrosis or late stages of apoptosis, when the metabolic activity is severely reduced (Franken *et al.*, 2006). Moreover, the surviving cells may be unable to proliferate because of reproductive integrity loss (Munshi *et al.*, 2005). Therefore, the cell ability to form a clone and produce a viable colony needs to be evaluated to measure the long-term cytostatic/cytotoxic effects of the tested agent (Franken *et al.*, 2006).

Once found that ester and amino were the least cytotoxic compounds as evidenced by XTT assay, and that in both cell lines cytotoxicity of ester and amino was detected from 62.5 μM, clonogenic survival was initially assayed in a concentration ranging from 2.5 to 80 μM, but at 40 and 80 μM no colony formation was detected. Moreover, in CHO-K1 no countable colony was detected after treatment with *cis*-TMS at all concentrations. The *trans-*TMS, ester and amino significantly reduced the colony formation starting at 5, 10 and 20 μM, respectively (Figure 3) in both cell lines. The ester was the least cytotoxic in this assay. Suppression of colony formation by stilbenes may be essentially irreversible, raising the possibility that interactions between stilbenes and the molecular entities and events they target may be permanent rather than transient above a certain threshold concentration (Hsieh *et al.*, 2011). Regarding the stereochemistry of the compounds, studies have shown that the *cis*-TMS is more cytotoxic than the *trans* isomer (Aldawsari and Velázquez-Martinez, 2015). According to Hsieh *et al.* (2011), experimental data about cellular signaling of PSA antigen in LNCaP cells, suggested that the stilbene core chemical structure instead of the side chain substitutions has a more critical role in controlling proliferative regulatory events.

Due to its high cytotoxicity, the *cis-*TMS was tested at lower concentrations in the clonogenic survival assay (Figure 4). Only at the 0.3125 μM concentration and lower it was possible to detected survival fractions in CHO-K1, while in HepG2 colonies were detect at 0.625 μM. Based on the results from this assay, the *cis*-TMS is almost 250-fold more cytotoxic than the amino and ester, and 128-fold more cytotoxic than *trans*-TMS. In the CBMN assay the most genotoxic compound was the *cis*-TMS, which induced a mean of 5.2 MN/100 cells at its highest concentration of 0.5 μM in CHO-K1 and 6.1 MN/100 cells in HepG2. This concentration was 5 times lower than the lowest concentration of the other stilbenes (Figure 5).

Both the cytotoxic and genotoxic effects of *cis*-TMS are in accordance with the results of *in vitro* studies reported in the literature. Schneider *et al.* (2003) demonstrated that the *trans*-TMS was 100-fold less effective in inhibiting the growth of Caco-2 cells than the *cis* isomer. Similar results were observed when *cis* and *trans* stilbenes were tested against five different cancer cell lines (Cushmann *et al.*, 1991). In the work of Paul *et al.* (2010), the *cis*-TMS was the most cytotoxic *in vitro* but it was not active *in vivo*. The lack of activity was associated with its low bioavailability. The *trans*-TMS significantly reduced tumor volume *in vivo*, showing much higher serum levels than the *cis-*TMS. Our results clearly demonstrated that the *cis-* is more genotoxic than *trans*-TMS and the other stilbenes tested. However, interestingly, CHO-K1 cells did not respond to *trans-*TMS treatment, while this stilbene was genotoxic in HepG2 cells, demonstrating the need for metabolization to exhibit genotoxicity. The *cis-*TMS also increased the frequency of MN, which occurs when a whole chromosome or a fragment forms from a chromosome break and is not incorporated into one of the daughter nuclei during cell division. Genotoxic agents can damage DNA directly or indirectly by interacting with non-DNA targets leading to genotoxic effects, essentially through lipid peroxidation and protein adducts (Kirsch-Volders *et al.*, 2003). As far as proteins are concerned, the literature reports inhibition of DNA repair enzymes, cell cycle control proteins, apoptosis-related gene products, nuclear lamins, defense proteins against oxidative damage, metabolization enzymes, and tubulins of the mitotic/meiotic spindle as mechanisms by which genotoxicants exert their effects (Kirsch-Volders *et al.*, 2003).

Schneider *et al.* (2003) demonstrated that the growth inhibition of Caco-2 cells by *cis*-TMS was not related to a cytotoxic effect, but it was associated with the arrest of the cell cycle progression at the G2/M transition phase. They postulated that the ability of a drug to block cells in G2/M is consistent with a disruption of the mitotic spindles by interaction with tubulin. The authors also demonstrated that *cis*-TMS inhibited tubulin polymerization in a dose-dependent manner.

Despite the biological promising effects of stilbenes, little is known about their genotoxicological effects. The present study showed for the first time, that ester, amino, *cis*- and *trans-*TMS stilbenes exhibit genotoxic effects. The *cis-*TMS is significantly more cytotoxic and genotoxic than the other stilbenes, and the genotoxicity of *trans*-TMS requires metabolic activation. Further studies may elucidate the mechanisms involved in the genotoxicity of the stilbenes evaluated in the present work.
